# RNASeqBrowser: A genome browser for simultaneous visualization of raw strand specific RNAseq reads and UCSC genome browser custom tracks

**DOI:** 10.1186/s12864-015-1346-2

**Published:** 2015-03-01

**Authors:** Jiyuan An, John Lai, David L A Wood, Atul Sajjanhar, Chenwei Wang, Gregor Tevz, Melanie L Lehman, Colleen C Nelson

**Affiliations:** Australian Prostate Cancer Research Centre - Queensland, Institute of Health and Biomedical Innovation, Queensland University of Technology, Princess Alexandra Hospital, Translational Research Institute, Brisbane, 4102 Australia; Queensland Centre for Medical Genomics, Institute for Molecular Bioscience, The University of Queensland, St. Lucia, 4072 Australia; School of Information Technology, Deakin University, 221 Burwood Highway, Burwood, VIC 3125 Australia

**Keywords:** RNA-seq, Genome browser, RNA secondary structure, SNP

## Abstract

**Background:**

Strand specific RNAseq data is now more common in RNAseq projects. Visualizing RNAseq data has become an important matter in Analysis of sequencing data. The most widely used visualization tool is the UCSC genome browser that introduced the custom track concept that enabled researchers to simultaneously visualize gene expression at a particular locus from multiple experiments. Our objective of the software tool is to provide friendly interface for visualization of RNAseq datasets.

**Results:**

This paper introduces a visualization tool (RNASeqBrowser) that incorporates and extends the functionality of the UCSC genome browser. For example, RNASeqBrowser simultaneously displays read coverage, SNPs, InDels and raw read tracks with other BED and wiggle tracks -- all being dynamically built from the BAM file. Paired reads are also connected in the browser to enable easier identification of novel exon/intron borders and chimaeric transcripts. Strand specific RNAseq data is also supported by RNASeqBrowser that displays reads above (positive strand transcript) or below (negative strand transcripts) a central line. Finally, RNASeqBrowser was designed for ease of use for users with few bioinformatic skills, and incorporates the features of many genome browsers into one platform.

**Conclusions:**

The features of RNASeqBrowser: (1) RNASeqBrowser integrates UCSC genome browser and NGS visualization tools such as IGV. It extends the functionality of the UCSC genome browser by adding several new types of tracks to show NGS data such as individual raw reads, SNPs and InDels. (2) RNASeqBrowser can dynamically generate RNA secondary structure. It is useful for identifying non-coding RNA such as miRNA. (3) Overlaying NGS wiggle data is helpful in displaying differential expression and is simple to implement in RNASeqBrowser. (4) NGS data accumulates a lot of raw reads. Thus, RNASeqBrowser collapses exact duplicate reads to reduce visualization space. Normal PC’s can show many windows of NGS individual raw reads without much delay. (5) Multiple popup windows of individual raw reads provide users with more viewing space. This avoids existing approaches (such as IGV) which squeeze all raw reads into one window. This will be helpful for visualizing multiple datasets simultaneously.

RNASeqBrowser and its manual are freely available at http://www.australianprostatecentre.org/research/software/rnaseqbrowser or http://sourceforge.net/projects/rnaseqbrowser/

**Electronic supplementary material:**

The online version of this article (doi:10.1186/s12864-015-1346-2) contains supplementary material, which is available to authorized users.

## Background

Genome browsers are necessary genomics tools as they enable visualization of multiple data simultaneously at a specific genomic locus. Recently, massive amounts of data have been produced from high-throughput microarray and next-generation sequencing (NGS) platforms. For example, the more commonly used NGS platforms (Illumina’s HiSeq and Life Technologies’ Ion Torrent) can produce gigabases of data per run [1]. Traditionally, the data generated by microarrays and NGS have been visualized at the candidate gene level using the UCSC genome browser [2]. The UCSC genome browser is currently the most commonly used tool and much public data can be found in their databases. Further, one’s data can be uploaded to examine it against these public datasets. However, there are limitations to the UCSC genome browser, some of which are inherent in its web-based application, such as the length of time to process large files (eg. BAM files).

The recently developed genome visualization tool, ZENBU [3], integrates transcript annotation with sequence analysis functions such as peak calling for ChIP-Seq and CAGE data, and normalization and quality filtering. However, ZENBU does not display individual raw reads, which is a valuable feature for biologists that are interested in splice variants. On the other hand, IGV [4] can display individual reads and all mapping attributes such as SNPs, InDels and customized Bed and Wiggle tracks, which is a very useful feature for biologists that enables them to simultaneously check multiple customized tracks in the same genomic region.

Thus, we have created RNASeqBrowser which is a stand-alone tool that accepts the UCSC genome browser BED and overlaid wiggle files [5], and was created using the platform independent Java computer language. Further, overlaying multiple wiggle data in one track is a much simpler process in RNASeqBrowser compared to the UCSC genome browser and other strand-specific genome browsers such as IGV [6], and Savant [7].

IGV is a useful and arguably the most widely used stand-alone genome browser. Thus, RNASeqBrowser has been designed to add more functionalities such as predicting secondary DNA/RNA structures using the VIENNA algorithm [8]. Furthermore, similar to the Tablet genome browser [9], the memory and CPU time consumption is displayed in the main window.

## Implementation

**Figure 1:**
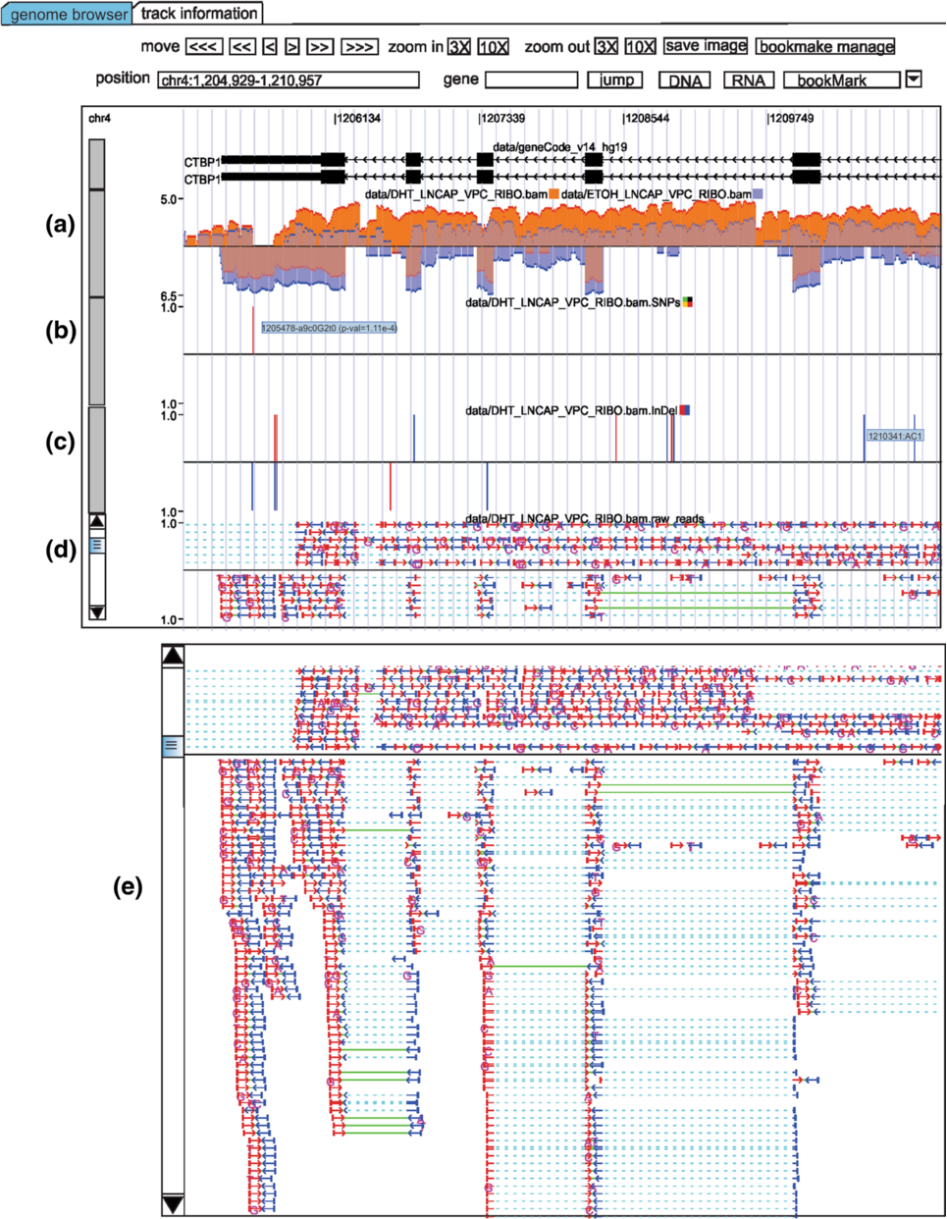
**Genome browser view showing the (a) wiggle track, (b) SNP track, (c) InDel track, (d) individual reads track, and (e) pop-up window for the individual reads.** All tracks have mouse over display functionality that shows detailed information such as transcript name, InDels’ sequences, SNP p-value and reads’ information.

Currently, most genome visualization tools [3,9-12] are modeled off the UCSC genome browser [13]. A features comparison of current genome browsers is listed in Table 1. These genome visualization tools need three types of information: (1) general genomic data such as the genome sequence and gene annotation. (2) initial setting information such as visualization screen size and which species is displayed in the view. (3) custom track information such as the wiggle file showing the coverage of sequencing data, or the Bed file showing the genomic region of interest. Genome sequence data is very big, therefore, in RNASeqBrowser, it is kept in a zipped format, and while gene annotation is in text format. RNASeqBrowser has two tabs: “genome browser” and “track information” tabs (Figure 1). The initial setting and track information can be added/deleted/modified in the track information tab.

**Table 1:** **Features comparison of current genome browsers**

**Track type**	**Tablet** **[** 9 **]**	**IGB** **[** 10 **]**	**IGV** **[** 4 **]**	**savant** **[** 11 **]**	**genomeView** **[** 12 **]**	**Ucsc genome browser** **[** 13 **]**	**ZENBU** **[** 3 **]**	**RNASeqBrowser**
bed		X				X		X
wiggle						X		X
bam wiggle	X	X	X	X	X		X	X
bam overlay wiggle					X			X
raw reads	X	X	X	X	X			X
raw read specific reads			X		X			X
paried end arrange			X		X			X
SNP calling				X	X		X	X
InDel				X	X			X
peak calling							X	
installation	S	S	S	S	S	W	W	S

S: stand alone.

w: web based.

### Genome browser

RNASeqBrowser’s genome browser comprises a reference transcriptome track and other custom tracks for the display of wiggle, bed, InDel and SNP data (Figure 1). Wiggle plots for transcripts that are expressed from either the positive or negative transcript strand are displayed above (positive strand transcription) or below (negative strand transcription) the central line (Figure 1a). Multiple wiggle plots can also be overlaid in one custom wiggle track. The advantage of overlaying multiple wiggle tracks is that it enables easy comparison of multiple datasets.

SNPs are called using the VarScan2 algorithm [14] based on the “MD” attribute in BAM files. SNPs are represented as a colored line (green for “A”-allele, red for “T”-allele, black for “C”-allele, and orange for “G”-allele), and the proportion of each allele that is expressed and the p-value can be shown by mousing over the SNP line (Figure 1b). The SNP calling uses the default VarScan2 parameters, although this can be modified by the user in the track information tab (see below). InDels are represented by a red (insertion) or blue (deletion) line and the inserted or deleted sequence can also be viewed by mousing over the InDel line (Figure 1c). Insertion information is obtained from the CIGAR string of the BAM file, and the deletion information is from both the CIGAR string and the deleted sequence in the “MD” attribute [5]. RNASeqBrowser can also display secondary DNA/RNA structure by clicking on the DNA or RNA button, respectively.

The individual reads track is a valuable track in RNASeqBrowser as it details the read strand, any mismatches, split reads, and paired reads for each mapped read (Figure 1d). A limited number of individual reads are embedded in the main browser window, but all reads can be viewed using the scrollbar to the left of the track. Read information such as ID, mapping location and read mate location can be viewed by mousing over each read. An optional popup window is also available to view all reads in one window Figure 1 (e). This window can be disabled by selecting the ‘hide’ option when adding BAM read tracks, or by editing the XML file in the track information tab.

Multiple exact read pairs are aggregated into one read pair, and the thickness of these read pairs represents how many reads are represented and the number of the exact same reads can be found in mousing over display. All reads or paired reads above the central line are from a positive strand transcript, and reads below the central line are from a negative strand transcript. The red (left to right) and blue (right to left) arrows represent the direction of the read. Paired reads are connected by a green line, with dashed lines representing a split read. Properly mapped reads are indicated by arrows pointing towards each other. Paired reads are plotted on the same level in the browser. RNASeqBrowser can also save the displayed genomic view in either EPS, PDF or SVG format using the ‘save button’.

### Track information

**Figure 2:**
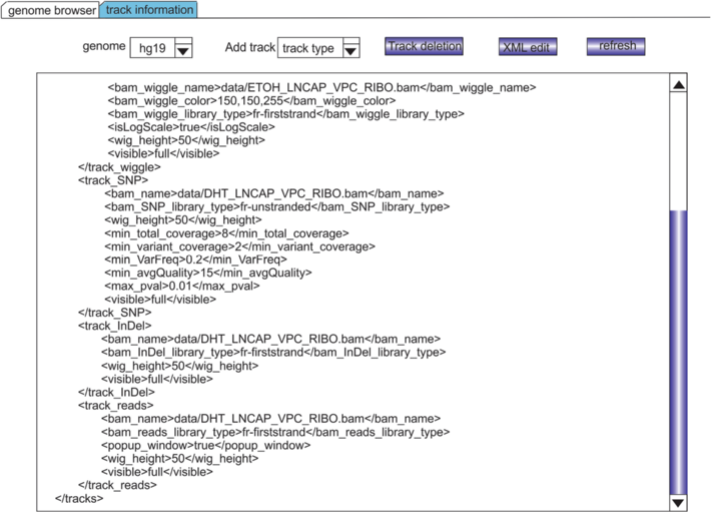
**Customize tracks.** XML is used to change all configurations and track display.

The XML format is employed in RNASeqBrowser to describe track files (Figure 2). Each track -- which contains the data file name and other information -- is enclosed by < track_xxx > and </track_xxx>. Tracks can be added/edited in the XML text box after clicking the “XML edit” button. Alternatively, users that are not familiar with the XML format can select “add track” or “track deletion” from the pull down menu button to add or delete tracks, respectively. Track changes are saved by simply clicking the refresh button which will also update the genome browser display.

RNASeqBrowser accepts many different file formats to output different visualization tracks (Additional file 1). Furthermore, visualization tracks such as wiggle plots can be generated using different input file formats. The track types and their corresponding input file types are as follows:

1. Reference track: Data can be downloaded from the UCSC table (http://genome.ucsc.edu/cgi-bin/hgTables?command=start). The default reference transcriptome track in RNASeqBrowser is Gencode v14. A user can add or change transcript track to Ensembl or Aceview annotations for the user preference. A reference transcriptome track, as its name suggests, is necessary to query a gene from the RNASeqBrowser interface in order to examine expression data that are input from BED, wiggle, or BAM files. Hence, reference track is considered as housekeeping track.
2. Bed tracks: RNASeqBrowser accepts UCSC genome browser Bed formats of 6, 9 and 12 columns. Bed tracks are displayed as per the UCSC genome browser. The reference track is a Bed track with 12 columns.
3. Wiggle tracks: RNASeqBrowser accepts UCSC wiggle files using the standard four column format: Chromosome name, start, end, and value. The value can be positive or negative to indicate the strand of the RNA. Display of wiggle tracks using UCSC genome browser files can be input using the “UCSC wiggle” option in the pull-down menu. RNASeqBrowser also accepts BAM files for direct and dynamic output of expression data into wiggle tracks. However, it is necessary for BAM files to be indexed (xxx.BAM.bai file) in order to maximize interface response from user queries. The BAM oriented tracks are generated from BAM files without the need for any intermediate files such as bedgraph (https://genome.ucsc.edu/FAQ/FAQformat.html). Display of wiggle tracks using sorted BAM files can be input using the “BAM wiggle” option in the pull-down menu. Implementing an overlay of multiple wiggle tracks in the UCSC genome browser within one track is a very involved process. Thus, RNASeqBrowser provides this functionality by simply inputting multiple UCSC wiggle or BAM files for an overlaid one track display in the genome browser.
4. SNP: RNASeqBrowser uses BAM files to determine SNPs and thus, does not require any intermediate files such as VCF (http://vcftools.sourceforge.net), which is usually generated by SNP calling algorithms such as GATK [15,16], to display SNP tracks. Since RNASeqBrowser intuitively shows the mismatch nucleotide to the reference genome, the SNP calling here adapted the VarScan [14] strategy. A position to make a call, the following criteria have to be satisfied: (1) minimum read depth (2)minimum number of reads with variant allele (3) minimum variant allele frequency (4) minimum base quality (5) max p value. These five parameters can be changed by the user in the “track information” tab in the interface. The SNP information is shown when mouse moving over the SNP line (Figure 1 (b)).
5. InDel: RNASeqBrowser uses BAM files to determine InDels. The InDel track shows insertions (red line) and deletions (blue line) from the RNAseq data (Figure 1 (c)).
6. Individual reads track: RNASeqBrowser uses BAM files to output the individual reads track. In the genome browser, the reads track has a scrollbar on the left side for a user to visualize all reads in the chromosome range. The information of a read, including read ID, read mapping location and mate chromosome location if it is PE, can be seen when mouse moving over on it. A popup window Figure 1 (e) for each read track is optionally provided for close study of read track.

Speed is one of the main issues for any genome browser tool. Consequently, BAM sequencing data has to be indexed before it is input into RNASeqBrowser. For reference, BED and UCSC wiggle files, RNASeqBrowser indexes all data files when first input. We used the most effective genome indexing strategy, Tabix [16,17], which results in an almost instantaneous retrieval of chromosome regions. In addition, the genome browser images are generated dynamically according to the genomic location, and then pasted onto the panel. This method results in much faster panel scrolling when compared with drawing figures directly onto a panel. Finally, every track corresponds to one image in the genome browser window for relocating tracks. Therefore, RNASeqBrowser only needs to replace images according to the tracks’ replacement without redrawing every image. However, visualizing large locus ranges can cause some display delays due to accessing of large amounts of genomic data.

To show the memory usage and CPU time consumed, on the main window title, the current used memory/total available memory and escaped time for the last query are appended. Compared to other genome tools such as Tablet and IGV, RNASeqBrowser used more memory and CPU time because RNASeqBrowser keeps SNP, InDel and coverage information dynamically calculated and is kept to save time for coming queries that are close to current query. Another reason is that RNASeqBrowser plots all paired reads at the same level in the canvas. To show 5 M reads, RNASeqBrowser takes about 500 M memory and 30 seconds CPU for normal PC.

As second generation sequencing projects increase sequencing depth, the window height required for plotting raw sequence reads rapidly increases. This results in huge memory requirements to store the information on the canvas, and long processing time to calculate read positions on the canvas. Our approach considers that large numbers of reads within a locus are likely to have the exact sequence reads. Thus, unlike IGV [4], RNASeqBrowser aggregates these exact reads into one, resulting in much less memory requirement and CPU processing time compared to IGV.

## Results and discussion

### Detecting chimeric transcripts

**Figure 3:**
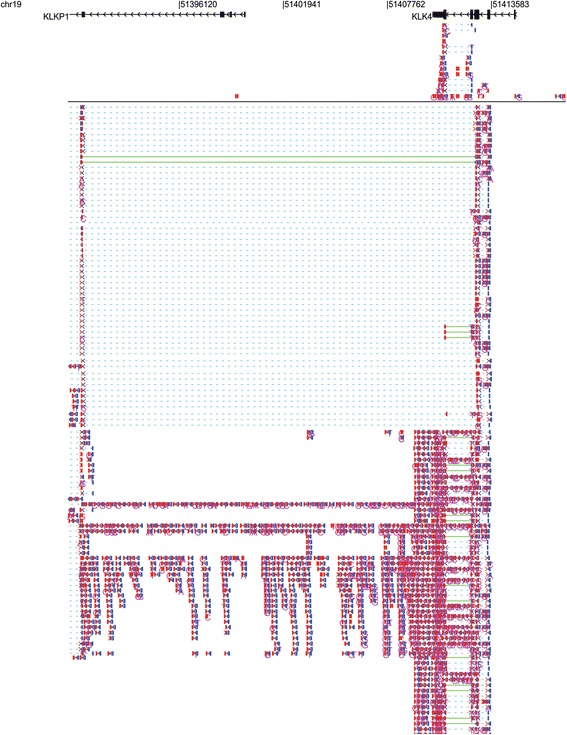
**Chimeric transcripts between KLK4 and KLKP1.**

Split reads represent RNA splicing that is distinct from the continuous DNA sequence. Thus, RNASeqBrowser can assist users to find chimeric transcripts and novel introns. Figure 3 shows a KLK4-KLKP1 chimeric transcript, whereby the third KLK4 exon and the fourth KLKP1 exon are spliced together. This chimeric transcript is supported by the large number of reads that are split between chr19: 51391131–51411834. This chimeric transcript has also been reported [18].

### Detecting and quantifying novel isoforms

**Figure 4:**
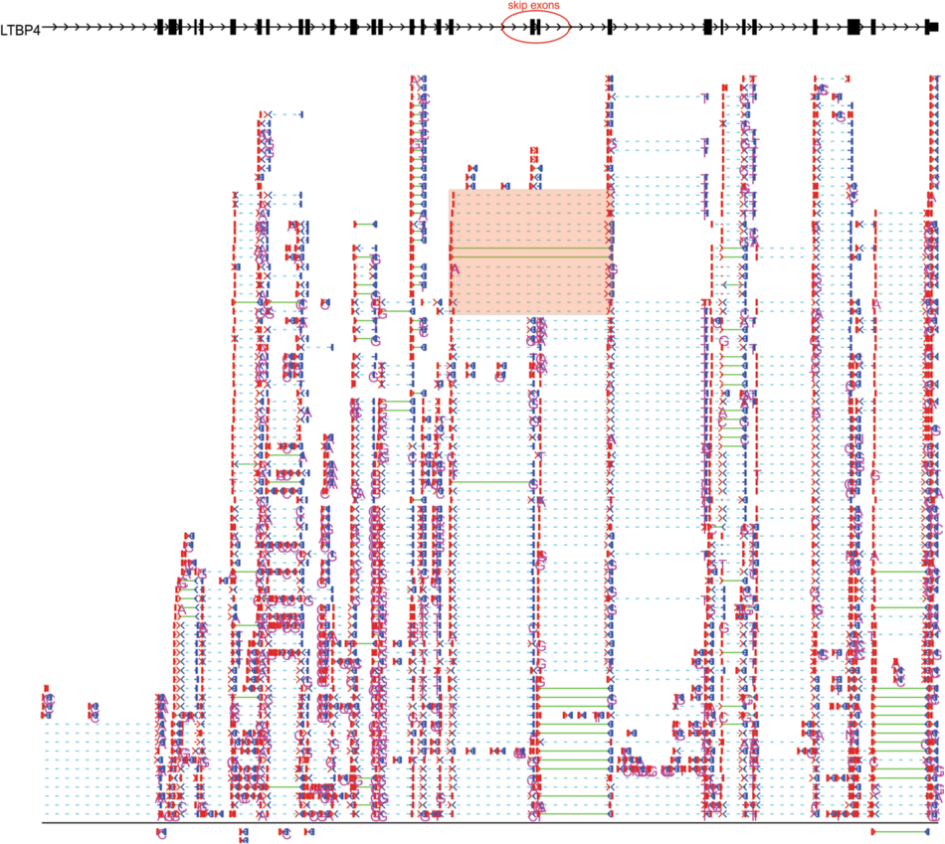
**Isoform identification from split reads.**

One of the limitations of wiggle plots is they are unable to identify novel splice sites for alternative transcripts. Although current RNAseq reads are not long enough to identify the whole transcript for most genes, RNASeqBrowser can identify splice-sites by displaying individual split reads at these splice junctions. Figure 4 shows a novel isoform for LTBP4 in prostate cancer cells (LNCaP). The individual reads window demonstrates that one third of the reads at this locus skip the two adjacent exons (Figure 4, orange box).

### Overlaid wiggles for differential expression analyses

**Figure 5:**
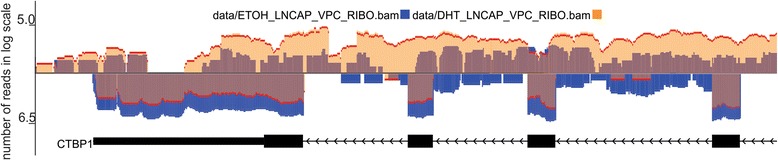
**Overlaid wiggles to show the differential expression.**

Wiggles represent gene expression at genomic loci. Therefore, it is useful to have a genome browser that can easily overlay multiple wiggle tracks from different conditions in the one track. This is highlighted in Figure 5, which shows the differential expression of the CIRBP gene (wiggle plot under line) and transcripts on the opposite strand (wiggle plot above the line) between mock (yellow wiggle) and androgen (green wiggle) treated LNCaP cells. Overlaid wiggle data for each treatment are displayed as brown wiggle plots on both the plus and minus strand. All wiggles have the same scale, so it will enable users to intuitively identify differential expression.

## Conclusion

In this paper, we introduce a strand specific RNAseq-oriented genome browser (RNASeqBrowser). RNASeqBrowser maintains the main functions of the UCSC genome browser (two main custom track types: Bed, bedGraph, can be uploaded into RNASeqBrowser), as well as adding four new track types (Individual reads track, SNP track, InDel track, and BAM wiggle). Coverage tracks (wiggle) and individual reads tracks have similar function to that of IGV, but the wiggle file in RNASeqBrowser can overlay multiple wiggle tracks into one track. The display of these new track types are all dynamically generated from BAM files without the need for any intermediate files. Thus, RNASeqBrowser should be easy to install and use for bioinformatic novices. In fact, the whole integration and use of RNASeqBrowser functionalities, such as the uploading of custom tracks using the graphics user interface, was designed to be user-friendly enough for general biologists to use. RNASeqBrowser’s individual reads track is also a useful feature for interrogating transcript structures, particulary for chimaeric transcripts and alternative exon usages. Further, data files that are commonly used in the UCSC genome browser are compatible with RNASeqBrowser. Finally, RNA/DNA secondary structures for genomic regions can be dynamically generated in a pop up window, extending the functionality of RNASeqBrowser over other useful strand-specific stand-alone genome browsers such as IGV.

## Availability and requirements

**Project name:** RNASeqBrowser.

**Project web page:**http://www.australianprostatecentre.org/research/software/rnaseqbrowser.

**Operating system(s):** Windows, Linux, Mac OS.

**Programming language**: Java.

**Other requirements:** JDK.

**License:** GNU General Public License.

**Any restrictions to use by non-academics:** None.

## Additional file

Additional file 1:
**RNASeqBrowser Manual.**
