# Supplementary material for: RNASeqBrowser: A genome browser for simultaneous visualization of raw strand specific RNAseq reads and UCSC genome browser custom tracks
Source: BMC Genomics. 2015 Mar 1;16(1):145. doi: 10.1186/s12864-015-1346-2 (PMC4355470; doi:10.1186/s12864-015-1346-2)
Supplement: Additional file 1: — RNASeqBrowser Manual. [file 12864_2015_1346_MOESM1_ESM.docx]

# RNASeqBrowser Manual

2015-01-05

Jiyuan An, John Lai, David Wood, Atul Sajjanhar, Chenwei Wang, Gregor Tevz, Melanie Lehman, Colleen Nelson: Australian Prostate Cancer Research Centre (APCRC-Q) and Institute of Health and Biomedical Innovation (IHBI), Queensland University of Technology (QUT), Brisbane, Australia

[j.an@qut.edu.au](mailto:j.an@qut.edu.au)

Web page: <http://www.australianprostatecentre.org/research/software/rnaseqbrowser>

Table of Contents

[RNASeqBrowser Manual 1](#_Toc408241501)

[1. Installation 2](#_Toc408241502)

[1.1. Java Requirement 2](#_Toc408241503)

[1.2. Start from web browser: 2](#_Toc408241504)

[1.2.1. Security issues 2](#_Toc408241508)

[1.3. Command Line start 3](#_Toc408241509)

[1.3.1 Start RNASeqBrowser 4](#_Toc408241510)

[1.3.2 Genome Browser 4](#_Toc408241511)

[1.3.2 Track Management 6](#_Toc408241512)

[1.3.2.1 Adding Track 6](#_Toc408241513)

[1.3.2.2 Deleting Track 17](#_Toc408241514)

[1.3.2.3 Editing track information 17](#_Toc408241515)

# Installation

## Java Requirement

RNASeqBrowser requires Java 6 or later to be installed in the computer. JDK (Java SE Development Kit) can be downloaded for installation from <http://www.oracle.com/technetwork/java/javase/downloads/index.html>

## Start from web browser:

| 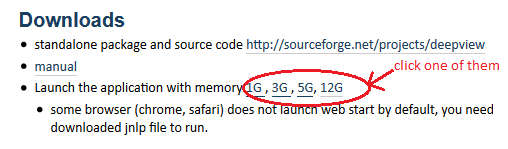 |
| --- |


## Security settings

- Some browsers (chrome, safari) do not launch web start by default; in this case, double-click the downloaded jnlp file to execute it.
- If jnlp file is not opened by "java web start", set "java web start" as the default application for "jnlp" file. Alternatively, RNASeqBrowser can be launched from the command line by typing:

>javaws http://data.australianprostatecentre.org/RNAseq/RNASeqBrowser/launch.jnlp

>javaws http://data.australianprostatecentre.org/RNAseq/RNASeqBrowser/launch_3G.jnlp

>javaws http://data.australianprostatecentre.org/RNAseq/RNASeqBrowser/launch_5G.jnlp

>javaws http://data.australianprostatecentre.org/RNAseq/RNASeqBrowser/launch_12G.jnlp

- If an error "Java Application Blocked" appears, a new DeploymentRuleSet.jar file needs to be installed in the computer in the following directories, or simply remove the "Deployment" folder.
- On Windows platforms, install the file in the *<Windows-directory>*\Sun\Java\Deployment directory, for example, c:\Windows\Sun\Java\Deployment.
- On Solaris or Linux platforms, install the file in the /etc/.java/deployment directory.
- On Mac OS X platforms, install the file in the /Library/Application Support/Oracle/Java/Deployment/ directory.
- In Mac, if an error "can't be opened from an unidentified developer" appears, go to System Preferences -> Security & Privacy -> (Unlock Window) -> Allow apps downloaded from: (Select Anywhere). The procedure is shown in Figure 1.

| 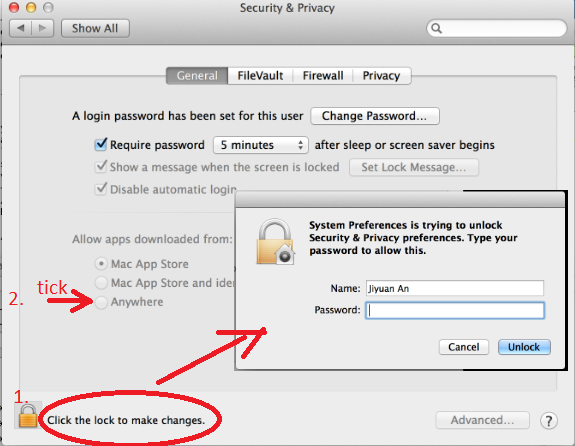 |
| --- |

Figure 1 security settings in Mac

## Command Line start

1. RNASeqBrowser related files are zipped at the URL: [http://www.australianprostatecentre.org/research/software/rnaseqbrowser](http://www.australianprostatecentre.org/research/software/rnaseqbrowser%20) .
2. Download the zipped file into a directory – e.g. C:\RNASeqBrowser (Windows), or /home/xxx/RNASeqBrowser (Linux).
3. Once the file is unzipped, a new directory “RNASeqBrowser” will appear. The directory contains four files:
   - **RNASeqBrowser.bat**, which is a batch file, which runs in a Windows environment. A shortcut can be created on the desktop to allow RNASeqBrowser to run by simply clicking the shortcut.
   - **RNASeqBrowser.sh** and

- **RNASeqBrowser.jar**, which stores java classes of RNASeqBrowser. This is the main file to run this tool.

And two subdirectories:

- - **Lib directory**, which contains all java libraries, used in RNASeqBrowser, such as RNAfold and picard libraries.
- **Data.** Under the data directory you will find:
- init.xml, which contains which genome will be visualized in the genome browser.
- Genome folders (hg19, hg18, mm10). Each folder has the following files.
- **Track.xml**, which contains information for the genome browser configuration and track. It is in XML (Extensible Markup Language) format. All items in the track.xml file, such as file name and colour, are enclosed within a pair of tags. In the beginning of XML file, the chromosome size of the target genome is listed. The tags, <locus> and <\locus>, are used to set the chromosome coordinates in the genome browser. For example:

*<locus>*

*<chr>chr19</chr>*

*<loci_start>51409009</loci_start>*

*<loci_stop>51414310</loci_stop>*

*</locus>*

- **Track files** such as BED, wiggle, and BAM files,
- **genomeData** which is zip file contains genome sequences. Genome sequences are fasta format and each chromosome corresponds to one fasta file.
- **Reference files** which have “tx” extension. Their index files (xxx.idx) will be created for their first time use.
- **Demo folder,** which contains demo track data files such as bam, bed and wiggle files.

# Start RNASeqBrowser

1. First ensure that java is executable, as in Figure 2. If not, you need to check the system variable path.

| 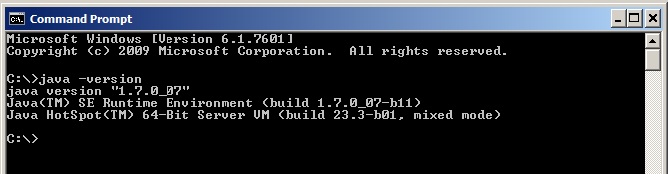 |
| --- |

Figure 2 test whether java is installed

1. In Windows, double click on the RNASeqBrowser.bat (800MB memory) or RNASeqBrowser_2G.bat (initializes 2GB memory) file to start RNASeqBrowser. You can also go to the command prompt to start RNASeqBrowser manually.
2. In Linux or MacOS, type: >java –jar –Xms2g RNASeqBrowser.jar , or .sh file. For example >./RNASeqBrowser_2G.sh, which initializes 2G memory for RNASeqBrowser.

The option –Xms sets the initial memory allocation pool in the machine. More memory is needed if a very deep RNAseq data is loaded. The memory and CPU usages are shown dynamically on the title of the RNASeqbrowser window.

RNASeqBrowser interface has two tabs: one for the genome browser window, a second for the track information window.

# Genome Browser

Once RNASeqBrowser has started, six tracks (as indicated in track.xml) are shown in the genome browser (Figure 3), where most buttons are functionally similar to that of the UCSC genome browser.

1. A track will be highlighted if the mouse pointer moves over on the left margin of the track.
2. Moving across the genome is achieved by pressing the <<<, or <<, or <,or >, or >>, or >>> buttons as follows:
   - < and > move the browser left or right by 10% of the genomic region currently displayed.
   - << and >> move the browser by 50% of the displayed genome, and
   - <<< and >>> move the browser by 100% of the displayed genome.
3. Zooming can be done with the 3X and 10X zoom in and out buttons, or by placing the mouse pointer at the top of the canvas of the genome browser, then clicking and holding the left mouse button. With the left mouse button held down, drag the pointer across to your locus of interest, and then release the left mouse button. This will enlarge the area of interest into the full canvas as shown in Figure 4. The highlighted region on the left is shown as the selected full-sized region on the right.
4. Tracks can be rearranged in the genome browser window. To reorder tracks, click and hold the left side of the track and drag the highlighted track to the desired new position.

| 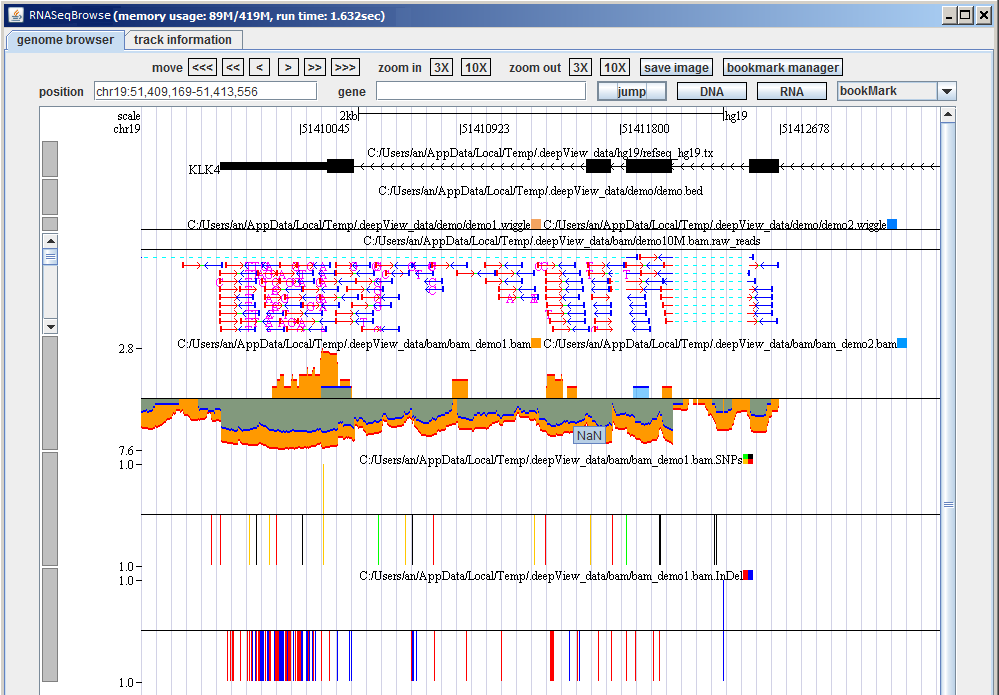 |
| --- |

Figure 3 View of the genome browser in RNASeqBrowser

1. A gene or locus can also be inputted in the “position” and “gene” textboxes. By clicking on the “jump” button, the desired locus is represented in full-size on the genome canvas.
2. The “bookmark manager” button may be used to mark loci that you are interested in for further use.
3. The “bookmark” button is used to select loci that you have marked previously.

| 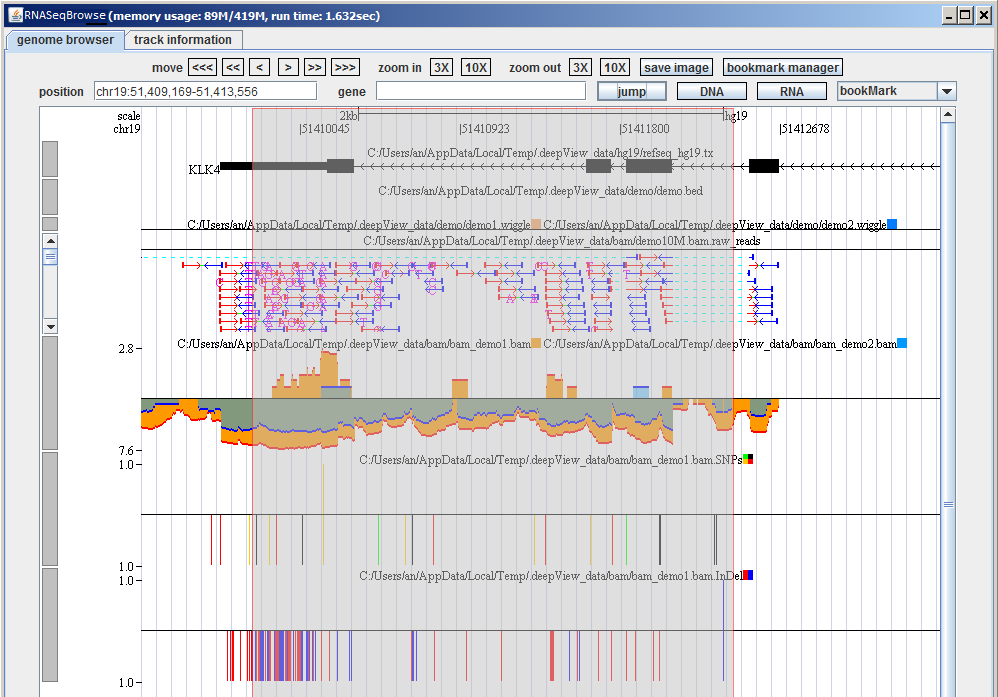 | 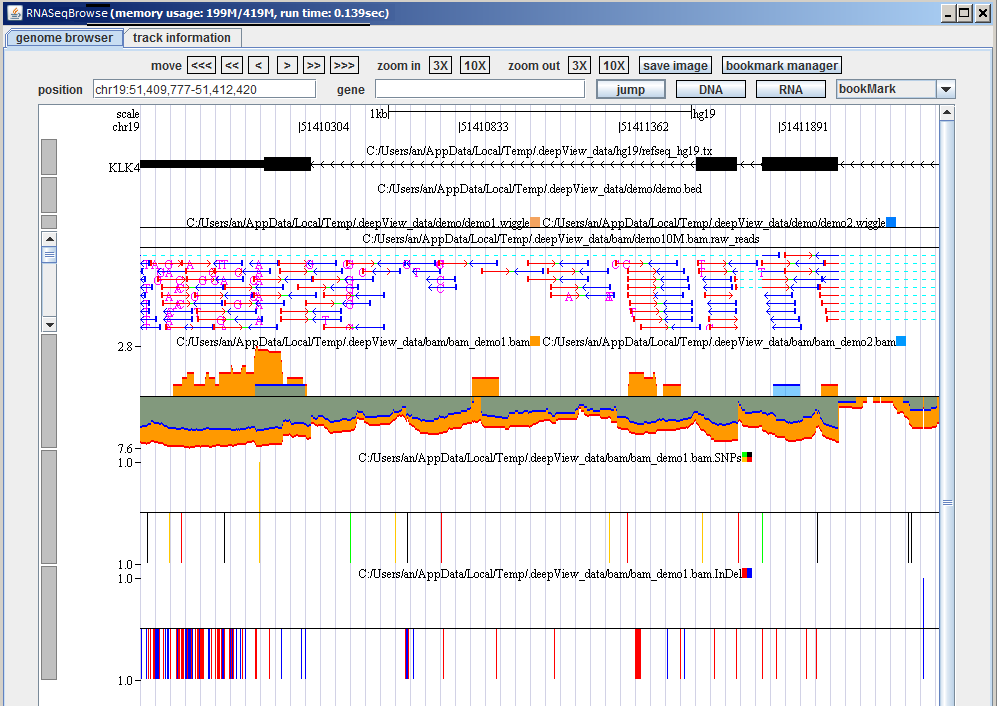 |
| --- | --- |

Figure 4 Zoom in by dragging the mouse

1. DNA and RNA buttons on the upper-right corner of the RNASeqBrowser browser may be used to obtain DNA sequence and RNA secondary structures in the genome browser window. The resulting sequence will be displayed in a pop up window as shown in Figure 5. The structure picture is presented as the Vienna RNAfold application tool with the secondary structure represented by brackets. The secondary structure figure is shown as well. The function is not supported in web version (unless users download hg19 genome data file “genomeData” from http://sourceforge.net/projects/rnaseqbrowser/files/data/hg19/ and put it under data/hg19/).

| 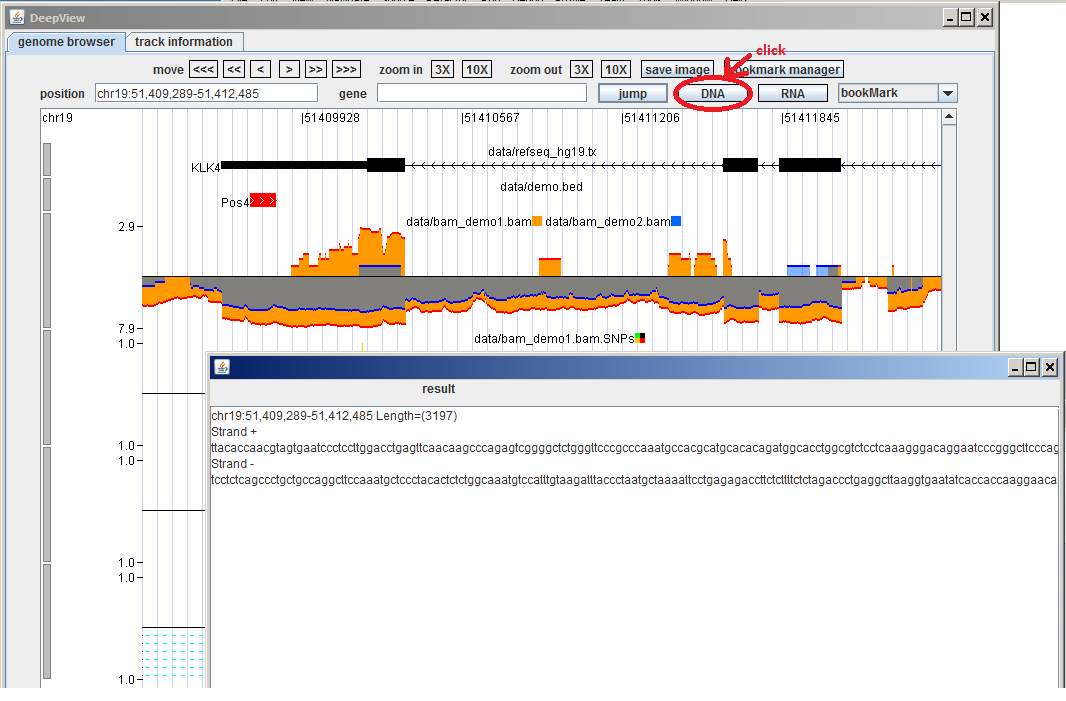 | **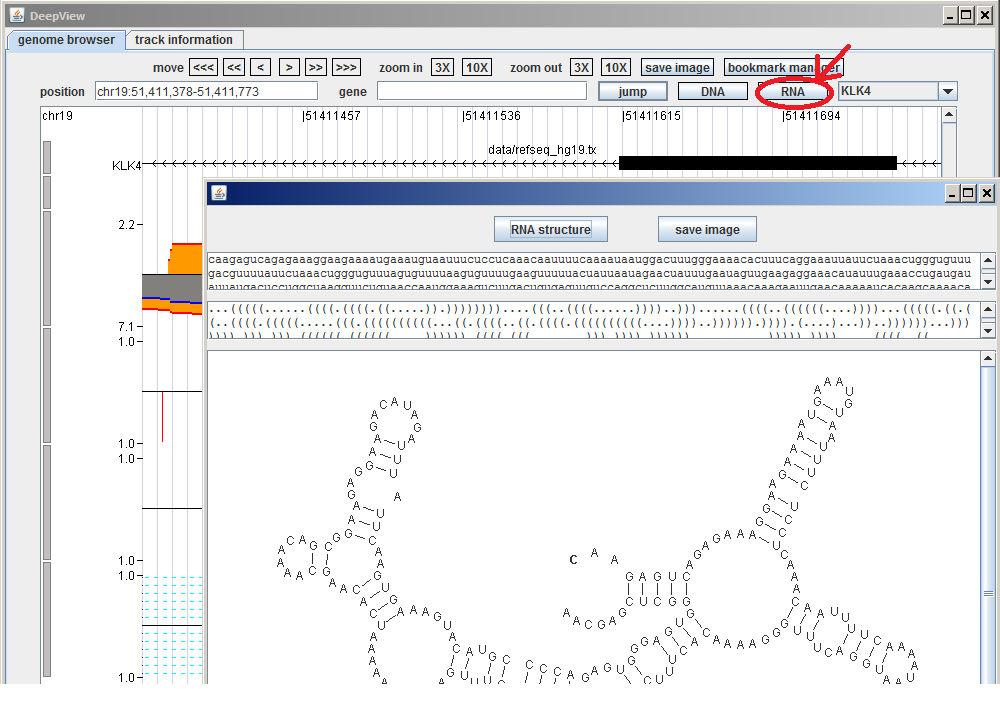** |
| --- | --- |

Figure 5 DNA and RNA secondary structure

# Track Management

# Adding Track

Users can add, remove, and edit tracks in a corresponding genome assembly using GUI as shown in Figure 6. In the download version, three genome assemblies were embedded (hg18, hg19, mm10). There are five items (Reference, Bed, UCSC wiggle, BAM wiggle and BAM reads) in the dropdown menu next to: “Add Track”. After selecting one item from the dropdown menu, an input dialogue pop-up appears (as shown in Figure 7).

Each Reference or Bed track corresponds to one data file. After choosing a Reference or Bed file and clicking “data file”, followed by “OK”, the track in XML format is appended in the textbox. The figure appears in “genome browser” tab, after clicking “refresh” button.

| 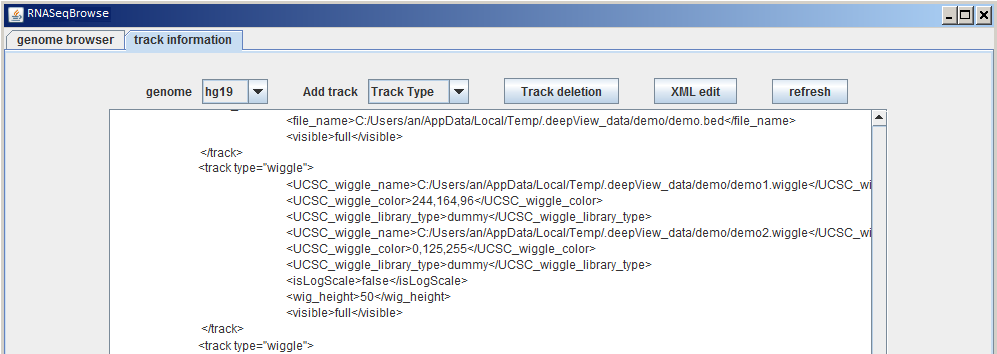 |
| --- |

Figure 6 customize tracks

RNASeqBrowser enables wiggle tracks with more than one wiggle file to be overlaid in a semi-transparent manner (“Add UCSC wiggle Track” and “add BAM wiggle Track” in Figure 7). In the wiggle track input dialogue, there is a “+” button to dynamically increase wiggle file. To compare wiggles, the representations of different wiggle files may be set to appear in contrasting colours. Similarly, this can be done for BAM wiggle file.

The only difference between two types of wiggle is that the UCSC wiggle track uses UCSC format, while BAM wiggle uses the indexed BAM file as input file.

The BAM track has three types: raw reads, SNP and InDel. The format of all their input files is indexed within the BAM file.

| 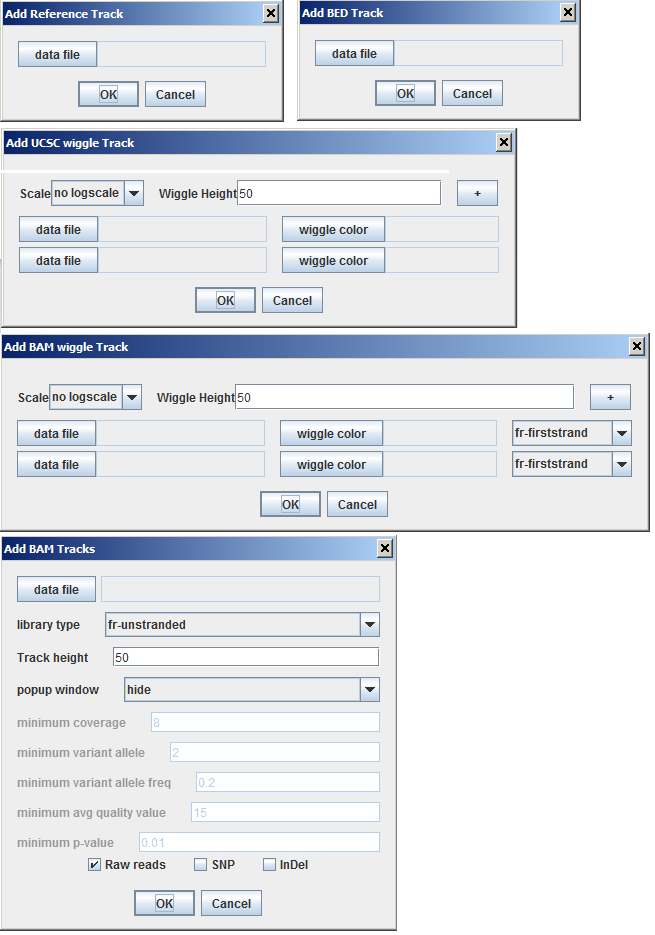 |
| --- |

Figure 7 track input interface

1. **Reference track**

Users can add Reference tracks, which represent transcript annotation, such as Refseq, Ensembl, Genecode, and Aceview. These transcript files can be downloaded from UCSC genome browser web page. The files should have the extension “.tx”.

Figure 7 shows all input interfaces for the “add track” process. In general, you need to create a reference track from a transcript annotation for the target genome. This has two functions:

• To make a gene symbol query from the genome browser.

• To indicate genes present in the locus of the genome browser.

The reference track is described in track.xml as shown:

<track_reference>

<file_name>geneCode_V12_hg19.tx</file_name>

<visible>full</visible>

</track_reference>

The tag <file_name> is followed by a transcript annotation file that is a text file with “.tx” as its extension. It is downloaded from UCSC genome browser. There are 15 columns, as listed below. The other annotation files can be downloaded from http://genome.ucsc.edu/cgi-bin/hgTables

a. #bin

b. Name

c. Chrom

d. Strand

e. txStart

f. txEnd

g. cdsStart cdsEnd

h. exonCount

i. exonStarts

j. exonEnds

k. score

l. name2

m. cdsStartStat

n. cdsEndStat

o. exonFrames

1. **BED track**

The UCSC genome browser BED format is adopted.

Six, nine, and 12 column formats are supported in RNASeqBrowser.

The first line contains column headings. Below is an example of the BED track with demo data:

*chrom chromStart chromEnd name score strand thickStart thickEnd itemRgb blockCount blockSizes blockStarts*

*chr19 51409723 51409828 Pos4 0 + 51409723 51409828 255,0,0*

*chr19 51412723 51412838 Neg1 0 - 51412723 51412838 0,0,255*

The definition of the columns is the same as the UCSC genome browser, namely:

1. **chrom** - The name of the chromosome (e.g. chr7, chrX, chr2_random).
2. **chromStart** - The starting locus of this item. The first base in a chromosome is numbered 0.
3. **chromEnd** - The end locus of this item. The chromEnd base is not included in the display of the feature. For example, the first 100 bases of a chromosome are defined as chromStart=0, chromEnd=100, and the display spans the bases numbered 0 to 99.
4. **name** - The name of this item. This label is displayed at the top of the BED line in the genome browser canvas. When the mouse hovers over the item for a few seconds, the name will appear.
5. **score** – This is not used in the RNASeqBrowser tool.
6. **strand** - Defines the strand as either '+' or '-'.
7. **thickStart** - The starting position at which the feature is drawn thickly (for example, the start exon in the gene display).
8. **thickEnd** - The end position at which the feature is drawn thickly (for example, the last exon in the gene display).
9. **itemRgb** - An RGB value of the form Red, Green, Blue (e.g. red colour: 255,0,0).
10. **blockCount** - The number of exons in the BED line.
11. **blockSizes** - A comma-separated list of the exon sizes. The number of items in this list should be the same as the blockCount.
12. **blockStarts** - A comma-separated list of block starts. All of the blockStart positions should be calculated relative to chromStart. The number of items in this list should be the same as blockCount.

The tag <file-name> is used for BED file name. In the XML file, <track_bed> tag is used as follows: *<track_bed>*

*<file_name>demo.bed</file_name>*

*<visible>full</visible>*

*</track_bed>*

Figure 8 shows how the BED track is shown in the genome browser and its setting in track.xml.

| 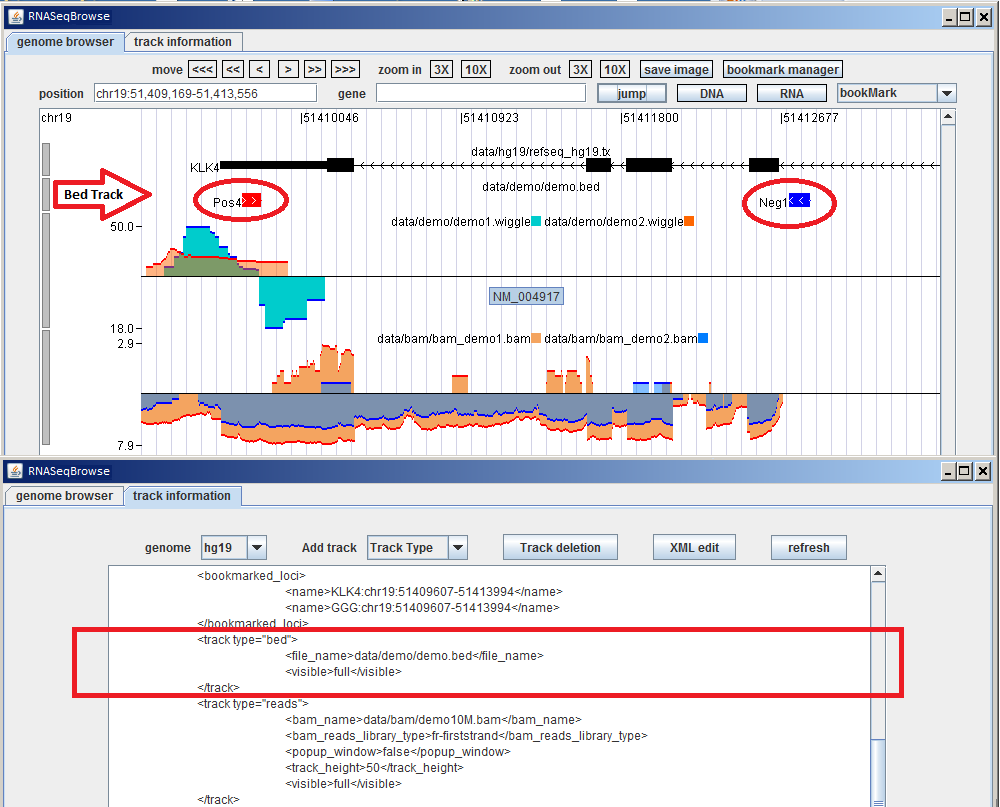 |
| --- |

Figure 8 BED custom track

1. **Wiggle track**

This is the same as the BedGraph track format in the UCSC genome browser:

*chr19 51409710 51409730 10*

*chr19 51409730 51409800 8*

*chr19 51409800 51409820 7*

*chr19 51402000 51402050 -10*

*chr19 51402050 51402150 -18*

There are four columns in the wiggle track:

1. **chrom** - The name of the chromosome (e.g. chr7, chrX, chr2_random).
2. **chromStart** - The start of the locus for this item. The first base in a chromosome is numbered 0.
3. **chromEnd** - The end of the locus for this item. The chromEnd base is not included in the display of the feature. For example, the first 100 bases of a chromosome are defined as chromStart=0, chromEnd=100, and the display spans the bases numbered 0 to 99.
4. **value** - the expression level. This corresponds to the height of the bar in the genome browser canvas. Positive values are represented by “+” strand and negative values are represented by “-”. These will be displayed in the genome browser canvas when the mouse pointer hovers over this position.

Figure 9 shows two wiggle files are semi-transparently overlaid into one track. The description to show overlaid wiggle files is as follows:

*<track_wiggle>*

*<UCSC_wiggle_name>demo1.wig</UCSC_wiggle_name>*

*<UCSC_wiggle_color>244,164,96</UCSC_wiggle_color>*

*<UCSC_wiggle_name>demo2.wig</UCSC_wiggle_name>*

*<UCSC_wiggle_color>0,125,255</UCSC_wiggle_color>*

*<isLogScale>false</isLogScale>*

*<wig_height>50</wig_height>*

*<visible>full</visible>*

*</track_wiggle>*

The tag <UCSC_wiggle_name> encloses the wiggle file name. The colour RGB 244,164,96 is given for the wiggle. The second wiggle file is described in the same way.

If tag <isLogScale> is set as “true”, the value lies in the natural log scale. Otherwise the original value is used.

The tag <wig_height> shows the height of the track. The tag <visible> is set to “full”.

| 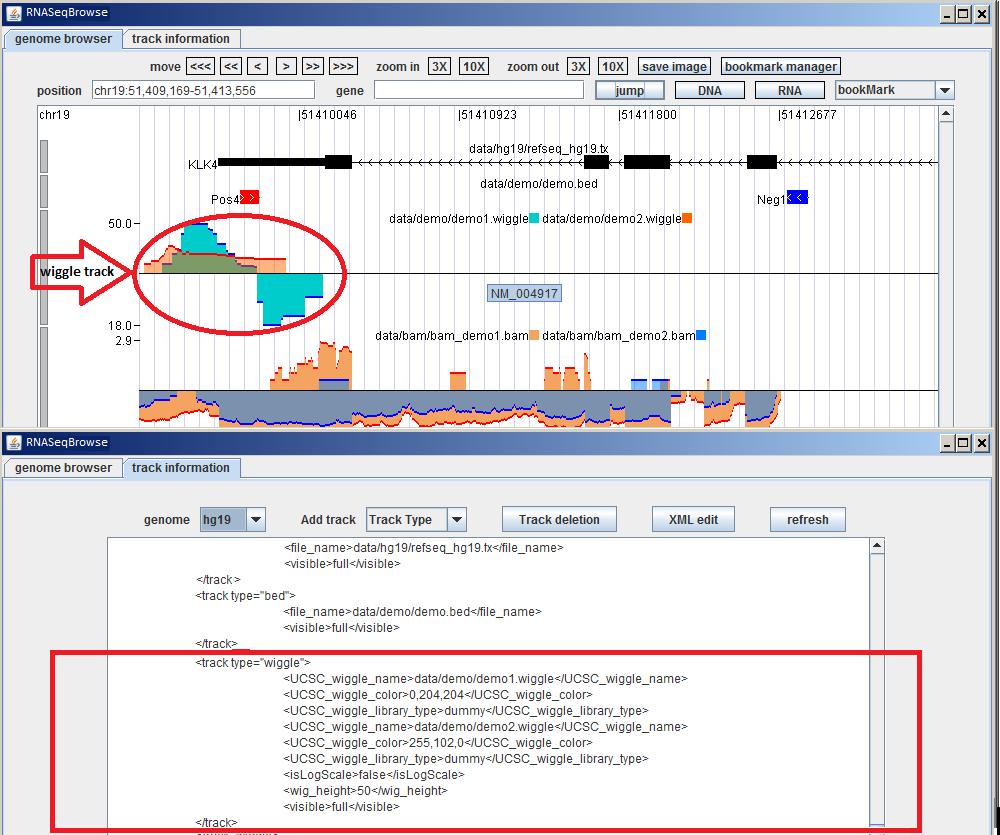 |
| --- |

Figure 9 How to add a wiggle track.

The top track of the genome browser canvas shows the added demo_wiggle track. Mousing over on the edges of the wiggle shows the value of a wiggle item.

1. BAM track

RNASeqBrowser produces three types of track for the BAM file:

1. **Wiggle (coverage)**

The genome coverage is calculated from this BAM file.

In the XML file, the BAM file name is set by tag <bam_wiggle_name>. The colour is set by tag <bam_wiggle_color>. The two tags can be repeated multiple times.

The wiggles generated by these BAM files will be overlaid as wiggle tracks described in Section 2.3.2. The tag <isLogScale> determines whether the values lie in the natural logscale. The remaining two tags <wig_height> and <visible> are the same as for the wiggle track.

Figure 10 shows the two BAM files bam_demo1.bam and bam_demo2.bam.

*<track_wiggle>*

*<bam_wiggle_name>bam_demo1.bam</bam_wiggle_name>*

*<bam_wiggle_color>244,164,96</bam_wiggle_color>*

*<bam_wiggle_name>bam_demo2.bam</bam_wiggle_name>*

*<bam_wiggle_color>0,125,255</bam_wiggle_color>*

*<isLogScale>true</isLogScale>*

*<wig_height>50</wig_height>*

*<visible>full</visible>*

*</track_wiggle>*

| 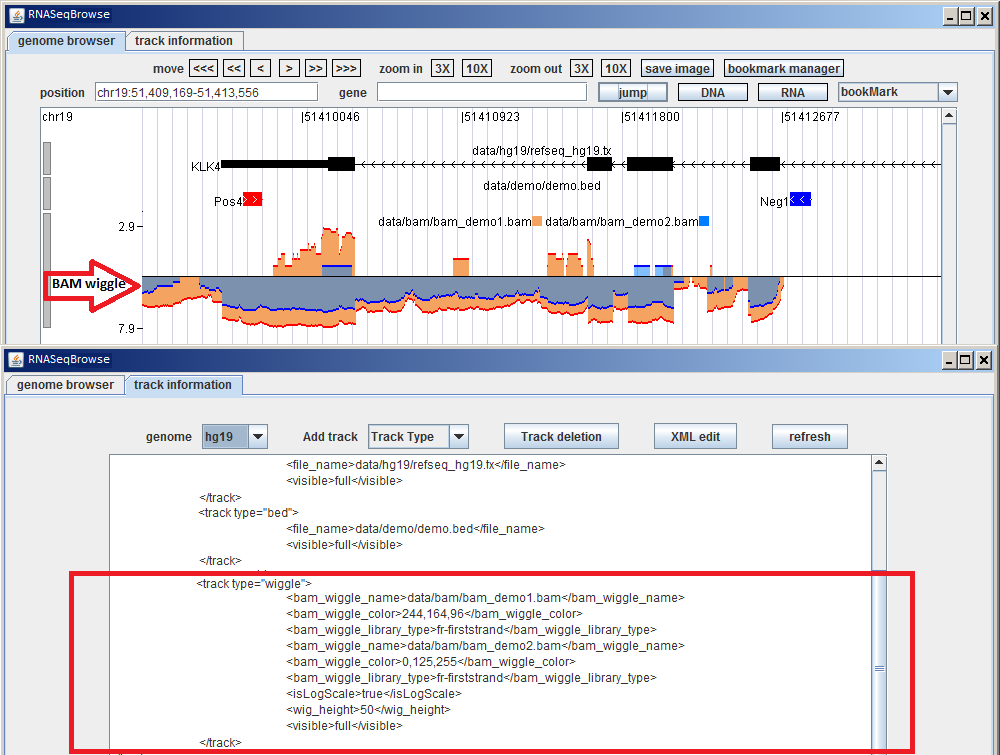 |
| --- |

Figure 10 Two demo BAM files comprising a bam_wiggle track.

1. **Raw reads**

The raw reads track (Figure 12) shows all reads aligned in the genome – using arrows to indicate read direction.

Red arrows indicate that the read’s direction is from left to right; while blue arrows show that the read’s direction is from right to left. For properly mapped, paired reads, one read should be from left to right and other should be from right to left in Transcript direction. In order to reduce memory requirements and to compress the pop-up window for the ‘raw reads’ track, paired reads that have the exact sequence are only shown once. However, the thickness of the arrow represents how many reads are represented for those multiple reads. For example, 1 to 2 reads are represented with 1 line in the arrow (bottom arrow in Figure 11 below), while 3 to 10 reads are represented by 2 lines in the arrow (middle arrow in in Figure 11 below), and more than 10 reads are represented by 3 lines in the arrow (top arrow in in Figure 11 below).

| 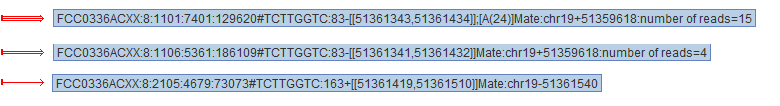 |
| --- |

Figure 11 mark of reads

The green solid line connects the paired reads that are properly mapped. The green dotted line indicates that a read is split in the genome.

The track height can be selected in the input interface. Since the number of raw reads mapping to the same chromosome loci usually amount to several hundreds, (sometimes, even tens of thousands), RNASeqBrowser provides a scrollbar on the left side of track. For extreme high expressed region, user can make a popup window with scrollbar, which allows the user to check every single read as shown in Figure 13. The description for raw reads is:

*<track_reads>*

*<bam_name>data/bam_demo1.bam</bam_name>*

*<bam_reads_library_type>fr-firststrand</bam_reads_library_type>*

*<popup_window>false</popup_window>*

*<track_height>50</track_height>*

*<visible>full</visible>*

*</track_reads>*

The library type is used to determine the transcript direction. The popup window showing reads is hidden by default. Users can edit to ‘true’ in <popup_window> by clicking the ‘XML edit’ button, or by selecting ‘show’ (‘hide’ in default) in the dropdown menu in the subfigure of Figure 7 when uploading the ‘bam reads’ track. Track height determines how many reads will be displayed in the track. Every read has a “mouse-over” display function. The display consists of read name, SAM flag, and its alignment loci followed by mate read coordinates.

| 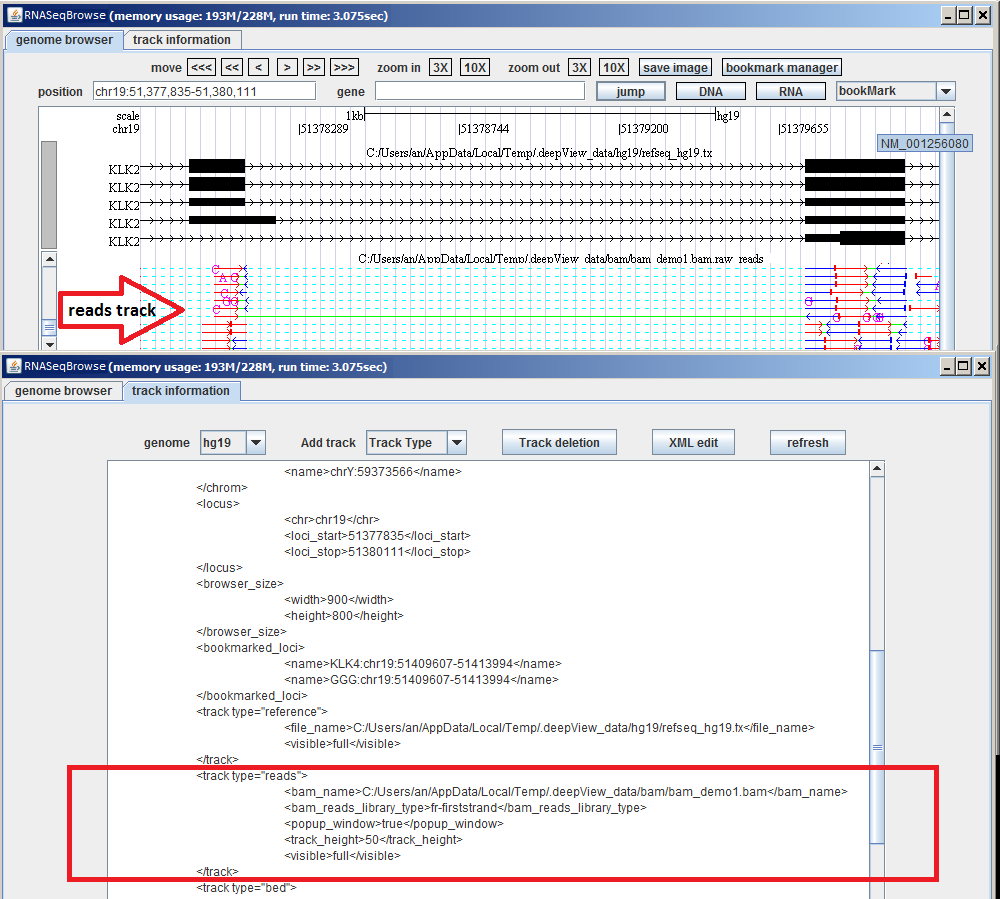 |
| --- |

Figure 12 Raw reads plot

| 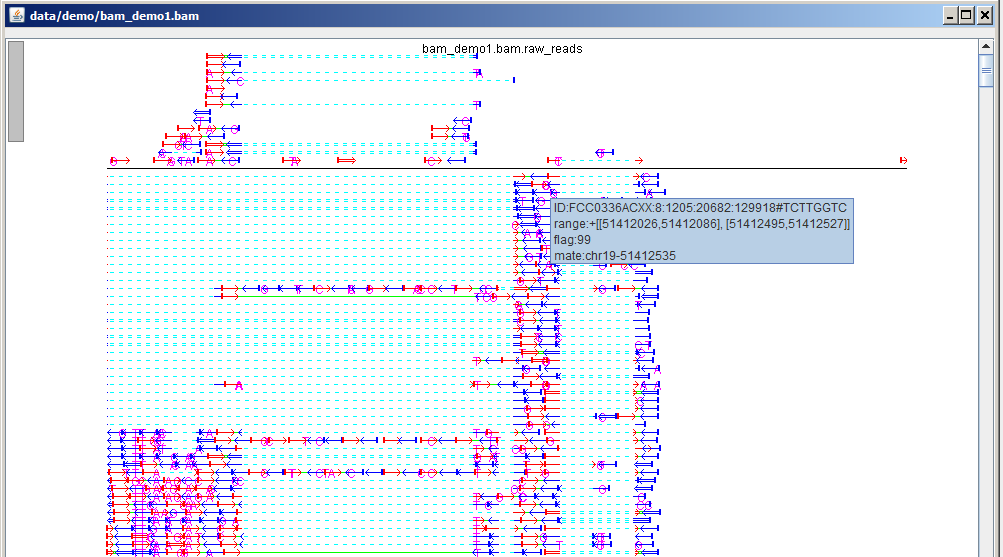 |
| --- |

Figure 13 raw reads window

1. **SNP track (mismatch of single nucleotide) (SNPs)**

SNP information is extracted from the “MD” attribute in the BAM file.

Bars with different colours denoting different genomic nucleotides (for example: “A”: green, “C”: black, “G”: yellow, and “T”: red) are displayed in the SNP positions.

The determination of SNP or mismatch is based on the individual expression of read; therefore, most SNPs only appear in “+” or “-” strands because most of the genome regions are only expressions of one strand. The bam_SNP track is enclosed by the tag <bam_SNP_name>.

Figure 14 shows SNPs for BAM file bam_demo1. The description of the SNP track is *<track_SNP>*

*<bam_name>data/bam_demo1.bam</bam_name>*

*<min_total_coverage>8</min_total_coverage>*

*<min_variant_coverage>2</min_variant_coverage>*

*<min_VarFreq>0.2</min_VarFreq>*

*<min_avgQuality>15</min_avgQuality>*

*<max_pval>0.01</max_pval>*

*<bam_SNP_library_type>fr-firststrand</bam_SNP_library_type>*

*<wig_height>50</wig_height>*

*<visible>full</visible>*

*</track_SNP>*

*The SNP calling uses VarScan algorithm.*

Where the item “min_total_coverage” represents the minimum read depth of the SNP variant allele.

“min_variant_coverage” represents the minimum number of reads with variant allele.

“min_VarFreq” represent s the minimum ratio of the number of reads with variant allele to the number of reads with reference genome allele.

“min_argQuality” represents the min average base quality of the reads.

“max_pval” represents the maximum fisher exact distribution p-value for variant allele comparing to genome reference allele.

| 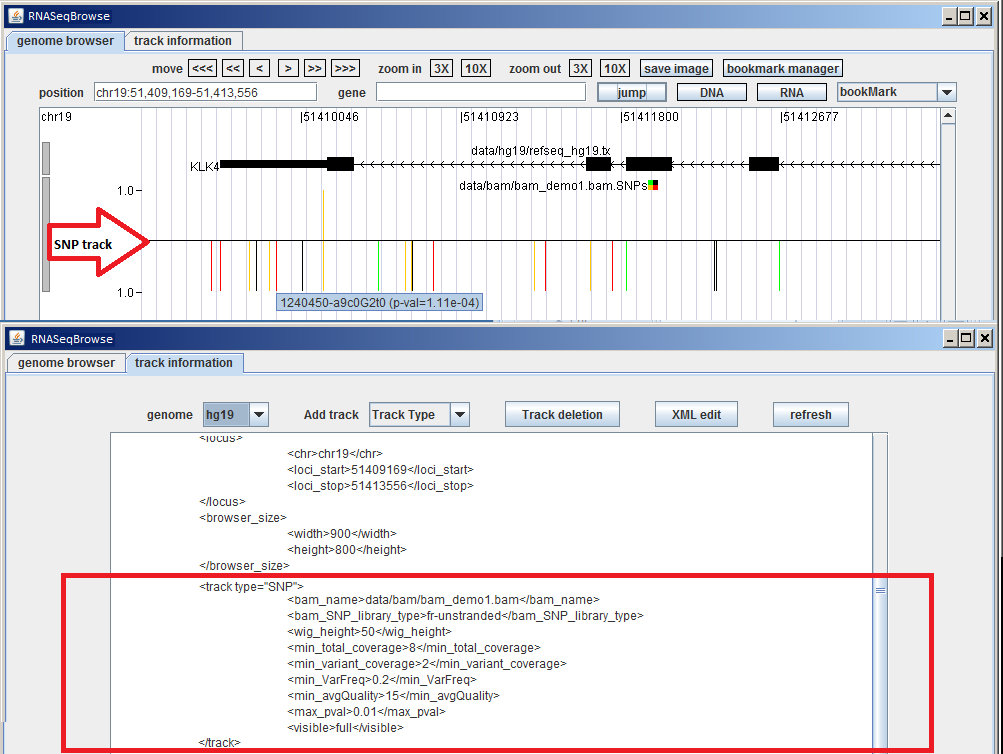 |
| --- |

Figure 14 SNPs for BAM file of a demo bam_SNP Track

1. **InDel track (insertion and deletion)**

The bam_InDel track is similar to the bam_SNP track. Insertion and deletion information is gathered from the Cigar flag and “MD” attributes of BAM files.

The tag <bam_InDel_name> encloses the BAM file from which the InDel information is extracted. The bar corresponding to the insertion position is red, and the bar corresponding to the deletion position is blue. The description in XML for the InDel is:

*<track_InDel>*

*<bam_name>data/bam_demo1.bam</bam_name>*

*<bam_InDel_library_type>fr-firststrand</bam_InDel_library_type>*

*<wig_height>50</wig_height>*

*<visible>full</visible>*

*</track_InDel>*

| 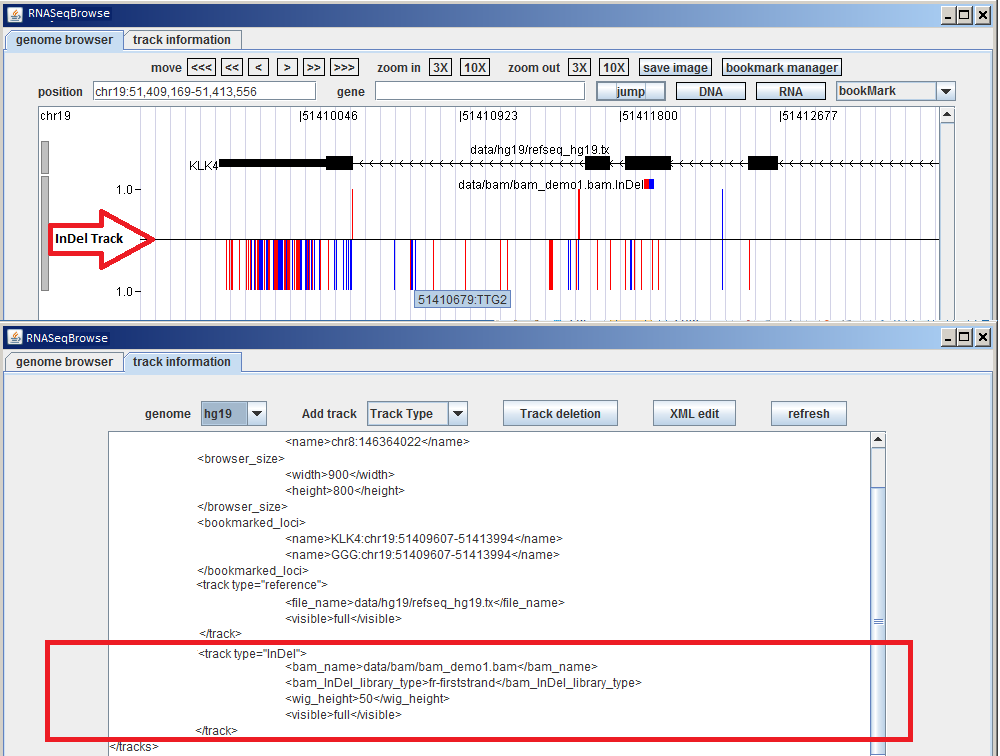 |
| --- |

Figure 15 A BAM_InDel track

# Deleting Track

A window showing the inputted tracks pops up when clicking “Track deletion” (popup shown in Figure 6). For Reference and Bed track types the listings in the popup window each start with track type, followed by the track file name. For other track types, the subtype of track appears before the tack file name.

Users can select the track type to be deleted by checking the box next to the relevant track name in the popup and then clicking the OK button.

If you want to restore the deletion, you need to click “refresh” to reload tracks.

| 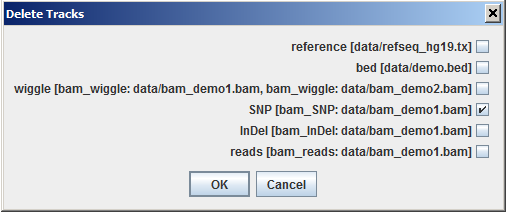 |
| --- |

Figure 16 track deletion

# Editing track information

The textbox is not editable before clicking “XML edit” (shown in Figure 6).

Each track corresponds to one closed XML item between <track_xxx> to </track_xxx>.

If a user is familiar with XML format, user can modify the XML text file directory without using the pop up dialogue box (shown in Figure 7). However, this should be done very careful, as the XML file may crash and RNASeqBrowser will not start up successfully, because of small typo or wrong XML format.
